# Dexmedetomidine for tracheal extubation in deeply anesthetized adult patients after otologic surgery: a comparison with remifentanil

**DOI:** 10.1186/s12871-015-0088-7

**Published:** 2015-07-23

**Authors:** Qing Fan, Chunbo Hu, Min Ye, Xia Shen

**Affiliations:** Department of Anesthesiology, The Eye, Ear, Nose and Throat Hospital of Fudan University, Shanghai Medical College of Fudan University, Shanghai, 200031 China

**Keywords:** Airway complications, Dexmedetomidine, Remifentanil, Tracheal extubation, Volatile anesthesia

## Abstract

**Background:**

Remifentanil and dexmedetomidine are well known to suppress airway reflexes during airway procedures. Smooth tracheal extubation is important after otologic surgery. The purpose of this study is to compare the effectiveness of dexmedetomidine or remifentanil infusion for producing smooth tracheal extubation in deeply anesthetized patients after otologic surgery.

**Methods:**

Seventy-four ASA I-II adult patients (18-60 years old) scheduled for elective otologic surgery were randomly assigned to one of three groups: sevoflurane-remifentanil (Group SR, *n* = 25), sevoflurane-dexmedetomidine (0.5 μg/kg) (Group SD5, *n* = 24), or sevoflurane-dexmedetomidine (0.7 μg/kg) (Group SD7, *n* = 25). Remifentanil or dexmedetomidine were administered for 10 min at the end of surgery. The primary outcome was the rate of smooth extubation. Respiratory pattern, airway obstruction, hemodynamic and respiratory profiles, time to awake, rescue analgesics in the post-anesthesia care unit (PACU), and postoperative nausea and vomiting (PONV) were also recorded.

**Results:**

The rate of smooth tracheal extubation as defined 1 min post-extubation was the same for Groups SR and SD7 (*P* > 0.05), but the rate of smooth extubation was lower for Group SD5 than for the other two groups (*p* < 0.05). During extubation, the respiratory rate was lower in Group SR than in both dexmedetomidine groups (*p* < 0.05). The hemodynamic profiles at extubation were similar between groups (*p* > 0.05), but the mean arterial pressure and heart rate were higher in Group SR at 10 and 15 min after extubation (*p* < 0.05). The incidence of airway obstruction and time to awake were comparable for all groups (*p* > 0.05). The need for rescue analgesic in the PACU was more common in Group SR than in both dexmedetomidine groups (*P* < 0.01). Compared to Group SR, both dexmedetomidine groups had less PONV on postoperative day 1 (*p* < 0.05).

**Conclusion:**

Combined with 1 MAC sevoflurane, dexmedetomidine 0.7 ug/kg and remifentanil provided similar rates for smooth tracheal extubation in spontaneously breathing, anesthetized adults. Dexmedetomidine exhibited opioid-sparing effects postoperatively and was associated with less PONV than remifentanil.

## Background

Deep extubation refers to the removal of a tracheal tube in a spontaneously breathing patient who is sufficiently anesthetized to obtund the laryngeal reflexes [1]. This technique offers the advantage of a smooth extubation with less airway stimulation, thereby reducing coughing, cardiovascular stimulation, and intraocular, intracranial, and middle ear pressure changes. Removal of the tracheal tube while patients remain deeply anesthetized may be advantageous in various situations [2]. It is particularly appealing for otologic surgery, as coughing can generate acute increases in pressure transmitted to the middle ear through the eustachian tubes, which may dislodge tympanic membrane grafts or disrupt other repairs. However, the risks of deep tracheal extubation include increased incidence of airway obstruction and aspiration.

Several strategies have been described to conduct a smooth extubation, including the use of a low dose remifentanil infusion [3–5]. In our previous study, we showed that the technique provided smooth extubation [5]. Remifentanil reduced the sevoflurane requirements by 30 % to maintain the same mean arterial pressure (MAP). However, the main disadvantages of remifentanil are its ultrashort duration of analgesia and risk of respiratory depression.

Because of its anxiolytic and analgesic qualities and lack of respiratory depressant effects, dexmedetomidine could be an attractive alternative. Although their clinical pharmacology and mechanisms of action differ, remifentanil and dexmedetomidine share similar properties, including suppression of airway reflexes [6–8] and reduction of sevoflurane requirements [9]. A single dose of dexmedetomidine (0.5 ug/kg) given before the end of the surgery was previously reported to attenuate airway and circulatory reflexes during extubation, without delaying recovery [10]. Consequently, we hypothesized that a single dose of dexmedetomidine would function like low dose remifentanil to facilitate a smooth extubation when used in combination with 1 MAC sevoflurane. We therefore conducted this prospective, randomized trial to compare the effects of remifentanil and dexmedetomidine when used during deep tracheal extubation in terms of the rate of smooth extubation, respiratory and hemodynamic profiles, recovery time, use of rescue analgesics in the post-anesthesia care unit (PACU), and incidence of postoperative nausea and vomiting (PONV) on the first postoperative day.

## Methods

After obtaining approval from the Hospital Ethics Committee (Shanghai Eye, Ear, Nose, and Throat Hospital affiliated with Fudan University) and written informed consent from each patient, we enrolled 75 adult patients who were 20-60 years of age and undergoing elective middle ear surgery between March 2013 and September 2013 (trial registry identifier, ChiCTR-TRC-13005025). All patients were American Society of Anesthesiologists (ASA) physical status I and II. Patients with chronic lung disease (e,g., emphysema or bronchitis), a current upper respiratory infection, asthma, mental disease, a smoking history, or other congenital diseases were excluded. Patients with hypertension, a known or suspected difficult airway (defined as a situation in which a trained anesthesiologist has difficulty performing facemask ventilation or tracheal intubation), a history of motion sickness, and an allergy to parecoxib were also excluded from the study.

All patients were kept *nil per os* for a minimum of 8 h before induction of anesthesia. They received no premedication. Standard ASA monitors were applied, and the end-tidal sevoflurane concentration was monitored after intubation. After inserting a 20-gauge catheter in an arm vein, anesthesia was induced with intravenous (IV) fentanyl 3 μg/kg and propofol 2.5 mg/kg, and mivacurium 0.2 mg/kg were administered to facilitate endotracheal intubation. Prior to the start of surgery, IV boluses of parecoxib 1.0 mg/kg, ondansetron hydrochloride 0.15 mg/kg, and dexamethasone 0.1 mg/kg were injected, and patients were randomly assigned to one of three groups according to a computer-generated random table until at least 25 patients were assigned to each group. For anesthesia maintenance, sevoflurane was adjusted to a MAC of 1.3, and patients received controlled ventilation with an inspired oxygen concentration of 0.35. At 30 min before the end of surgery, the patients were allowed to breathe spontaneously after reversal agents (neostigmine 0.07 mg/kg and atropine 0.02 mg/kg) were administered to prevent possible residual block and after return of neuromuscular function was confirmed using train-of-four peripheral nerve stimulation. Adequate spontaneous respiration was defined as a normal end-tidal carbon dioxide (ETCO_2_) waveform and an ETCO_2_ concentration less than 6.0 kPa.

At the end of surgery, sevoflurane was reduced to 1.0 MAC and the following infusions were administered for 10 min: remifentanil 0.03 μg/kg/min in Group SR, dexmedetomidine 0.5 μg/kg in Group SD5, and dexmedetomidine 0.7 μg/kg in Group SD7. During the infusion, patients breathed spontaneously and respiration was not assisted. Once the infusion was finished, the surgical assistant was allowed to apply the head dressing. The MAP and heart rate (HR) were maintained within 30 % of the preanesthetic baseline values in all groups throughout surgery. Any patient who could not maintain stable spontaneous respiration while deeply anesthetized was excluded.

An anesthesiologist, blinded to the assignment and with 12 years of clinical experience, performed the tracheal extubation using a standardized technique. After deflating the tracheal tube cuff, the oropharynx was suctioned before gently removing the tube. Sevoflurane was discontinued immediately after extubation. A nasopharyngeal tube was inserted and oxygen 8 L/min was administered via a facemask. The patient was maintained in the supine position. Smooth tracheal extubation was defined as no gross purposeful muscular movement, such as coughing, within 1 min of tracheal tube removal [11]. Patients with coughing, breath holding, or laryngospasm immediately after extubation were regarded as not having a smooth tracheal extubation. The extent of coughing was assessed using a 5-point scale: 1, no coughing; 2, minimal coughing (1 or 2 times); 3, moderate coughing (3 or 4 times); 4, severe coughing (5-10 times) and straining; and 5, and coughing > 10 times [12]. With the patient in supine position, the upper airway was assessed. Patients were categorized as having a patent or obstructed airway. A patent airway was defined as an SpO_2_ > 97 % while receiving 100 % oxygen and the presence of clear breath sounds, normal chest wall movement, and a normal ETCO_2_ waveform (obtained with a sampling cannula located in the facemask). Chin-lift was performed by the anesthesiologist if airway obstruction occurred. An investigator blinded to the treatment assessed the smoothness of extubation, respiratory pattern, and presence of respiratory complications. A regular respiratory pattern was defined as a stable respiratory rate (measured by ETCO_2_ monitoring) and a normal ETCO_2_ waveform. Respiratory and hemodynamic profiles were recorded before anesthesia, start of infusion, at extubation, and at 1, 5, 10, and 15 min after extubation.

In the PACU, the time from extubation to awake (i.e., eye opening on verbal command) was recorded. Patients remained in the PACU until they achieved a modified Aldrete score of 9 or 10 [13]. A visual analogue scale (VAS) score from 0 to 10 (0 = no pain, 10 = worst pain imaginable) was used to assess the level of pain. If the VAS pain score was > 4, a rescue analgesic (IV morphine 0.15 mg/kg) was given. All patients were interviewed by an anesthesia research nurse on the first postoperative day (during their hospital stay or at home via telephone) regarding their level of pain and the presence of PONV.

In our previous study, we found that the incidence of smooth extubation was 90 % in adult patients receiving sevoflurane and remifentanil combination [14]. Based on an estimate of a 25 % reduction in the incidence of smooth extubation, we estimated that a sample of at least 22 patients per group would be required to detect a significant difference among groups at an alpha level of 0.05, with a power of 0.8. Twenty-five patients per group were enrolled to provide a potential loss of 10 % protocol violation. Continuous data were reported as mean ± SD. One-way analysis of variance and *post hoc* Bonferroni tests were used to compare differences among groups. Nominal data were analyzed using either *x*^2^ or Fisher’s exact tests. Interactions between time and group factors in a two-way ANOVA with repeated measurements were used to analyze differences in hemodynamic profiles (i.e., HR and MAP) between patients in the three groups. *Post hoc* Bonferroni tests were used to compare differences in hemodynamic variables among groups at specific times. *P*-values < 0.05 were considered statistically significant. The primary outcome measurement was the effect of dexmedetomidine and remifentanil on the rate of smooth extubation after otologic surgery. The secondary outcome measures were the respiratory and hemodynamic profiles during extubation, recovery time, VAS pain score, and incidence of PONV.

## Results

**Fig. 1:**
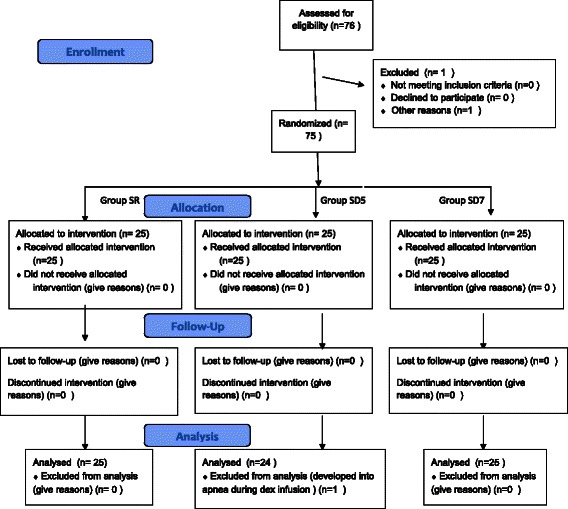
CONSORT flow diagram

A total of 76 patients were assessed, but surgery was cancelled for one patient. Thus, 75 patients were enrolled in the study. One patient in Group SD5 developed respiratory depression during the dexmedetomidine infusion and was excluded from the analysis. A total of 74 patients progressed through the study: Group SR (*n* = 25), Group SD5 (*n* = 24), and Group SD7 (*n* = 25) (Fig. 1). The demographics and other characteristics of the three groups are presented in Table 1. There were no significant differences between groups for age, weight, sex, duration of anesthesia, or dose of fentanyl.

**Table 1:** Demographics and clinical characteristics

Variable	Group SR	Group SD5	Group SD7	*P* value
	(*n* = 25)	(*n* = 24)	(*n* = 25)	
Sex (male/female)	15/10	13/12	14/11	0.32
Age (y)	42.3 ± 13.2	44.3 ± 14.3	40.0 ± 11.7	0.52
Weight (kg)	63.2 ± 10.0	61.4 ± 11.1	62.9 ± 11.0	0.82
Anesthesia duration (min)	109.0 ± 45.6	101.6 ± 31.0	109.5 ± 43.3	0.75
Dose of fentanyl (μg)	211.7 ± 40.3	216.5 ± 40.4	217.2 ± 42.0	0.84

Data are expressed as mean ± standard deviation (One-way ANOVA test) or number of patients (Chi Square test)

*SR* sevoflurane-remifentanil group, *SD5* sevoflurane-dexmedetomidine group (0.5 μg/kg), *SD7* sevoflurane-dexmedetomidine group (0.7 μg /kg)

In Group SR, 22 patients had a smooth extubation (no coughing on extubation), 1 patient had minimal coughing during extubation, and 2 patients had moderate coughing on extubation (Table 2). In Group SD5, 15 patients had smooth extubation, 6 patients had episodes of coughing (2 had minimal coughing, 4 had moderate coughing, and 2 had severe coughing, respectively), and 1 experienced partial laryngospasm, with a transient reduction in SpO_2_ to 81 %, which was treated with continuous positive airway pressure (CPAP). In Group SD7, 22 patients had smooth tracheal extubation and 3 patients had minimal coughing. The rate of smooth extubation was the same for Group SR and SD7 (*p* > 0.05). However, the rate of smooth extubation was significantly lower in Group SD5 (*p* < 0.05). No patient in Group SR or Group SD7 experienced desaturation, and the only patient who developed desaturation in Group SD5 was the individual who developed laryngospasm. No patient required re-intubation. The occurrence of airway obstruction was comparable in the three groups (*p* > 0.05). There was a tendency for more patients in Group SR and Group SD7 to maintain a regular respiratory pattern during dressing application, although the difference between groups did not reach statistical significance (*p* > 0.05). The percentage of patients with a regular respiratory pattern during cuff deflation was lower in Group SD5 (0.75, 95 % CI: 0.53-0.90) than in Group SD7 (0.88, 95 % CI: 0.69-0.97) and Group SR (1.00, 95 % CI: 0.86-1.00), *P* < 0.05.

**Table 2:** Extubation characteristics, respiratory complications, and respiratory pattern

Study group	Group SR	Group SD5	Group SD7	*P* value
	(*n* = 25)	(*n* = 24)	(*n* = 25)	
Smooth extubation	22 (88.0)	15 (62.5)	22 (88.0)	0.038
Coughing extent				0.096
No coughing	22	15	22	
Minimal coughing	1 (4.0)	2 (8.3)	3 (12.0)	
Moderate coughing	2 (8.0)	4 (16.7)	0	
Severe coughing	0	2 (8.3)	0	
Respiratory complications				
Laryngospasm	0	1 (4.2)	0	0.35
Desaturation (SpO_2_ < 90 %)	0	1 (4.2)	0	0.35
Airway obstruction	5 (20.0)	2 (8.3)	3 (12.0)	0.47
Regular respiratory pattern				
During dressing application	20 (80.0)	15 (62.5)	19 (76.0)	0.35
During cuff deflation	25 (100)	18 (75.0)	22 (88.0)	0.028

Data are expressed as number of patients (%) (Chi-Square test)

*SR* sevoflurane-remifentanil group, *SD5* sevoflurane-dexmedetomidine group (0.5 μg/kg), *SD7* sevoflurane-dexmedetomidine group (0.7 μg /kg)

At the time of extubation, the respiratory rate was significantly lower in Group SR (6.9 breaths/min) than in either dexmedetomidine group (Group SD5, 12.3 breaths/min; Group SD7, 12.2 breaths/min; *P* < 0.05) (Table 3). However, the respiratory rate in Group SR rose to a mean of 10 breaths/min at 5 min after discontinuing remifentanil. Tidal volume was higher in Group SR than in both dexmedetomidine groups (Group SR, 397.2 ± 136.9 ml; Group SD5, 275.7 ± 76.4 ml; Group SD7, 266.2 ± 70.3 ml; *P* < 0.05). ETCO_2_ was comparable among groups (*P* > 0.05).

**Table 3:** Respiratory profiles at extubation

Study group	Group SR	Group SD5	Group SD7	*P* value
	(*n* = 25)	(*n* = 24)	(*n* = 25)	
Respiratory rate (breaths /min)	6.9 ± 1.8	12.3 ± 5.2 ^^^^	12.2 ± 3.5**	<0.0001
Tidal volume (ml)	397.2 ± 136.9	275.7 ± 76.4^^^^	266.2 ± 70.3**	<0.0001
ETCO_2_ (mm Hg)	55.6 ± 10.3	52.3 ± 9.3	51.4 ± 8.6	0.26

Data are expressed as mean ± standard deviation

*SR* sevoflurane-remifentanil group, *SD5* sevoflurane-dexmedetomidine group (0.5 μg/kg), *SD7* sevoflurane-dexmedetomidine group (0.7 μg /kg)

**P < 0.001 (One-way ANOVA test), Group SD7 compared to Group SR ^^^^*P* < 0.001 (One-way ANOVA test), Group SD5 compared to Group SR

**Fig. 2:**
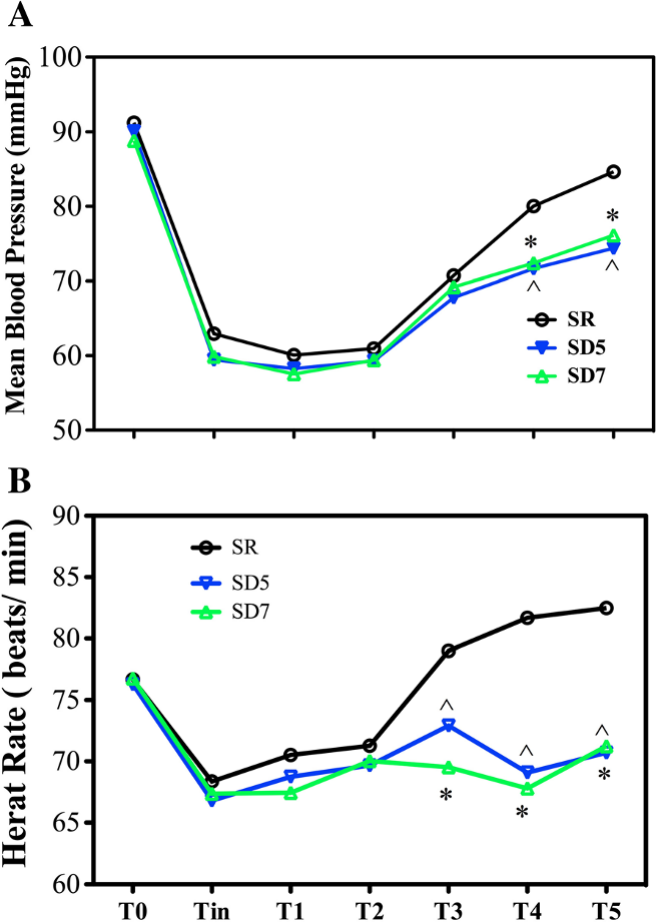
Hemodynamic changes in the three groups. T0 (before anesthesia); Tin, start of infusion; T1, at the time extubation; T1, 1 min after extubation; T2, 5 min after extubation; T3, 10 min after extubation; T4; 15 min after extubation. ^*p* < 0.05, Group SD5 compared to Group SR; *p < 0.05, Group SD7 compared to Group SR. Changes in MAP (**a**) and HR (**b**) in three groups at different perioperative time points

MAP was comparable between groups before anesthesia, start of infusion, at extubation, and at 1 and 5 min after extubation (Fig. 2). However, MAP was significantly higher at 10 and 15 min after extubation in Group SR than in the dexmedetomidine groups (*P* < 0.05). HR was higher in Group SR than in the dexmedetomidine groups at 5, 10, and 15 min after extubation (*P* < 0.05) (Fig. 2).

In the PACU, no patient developed oxygen desaturation. One 18-year-old male in Group SR exhibited agitation, which was treated with propofol and morphine. The time from extubation to awake (eye opening on verbal command) was comparable in the three groups (*p* > 0.05). However, more patients in Group SR required morphine for analgesia rescue (*p* < 0.001). The incidence of PONV (1 or 2 on the PONV scale) was lower in Group SD5 and Group SD7 than in Group SR (*p* < 0.05) (Table 4).

**Table 4:** Recovery variables in the post-anesthesia care unit and on postoperative Day 1

Study group	Group SR	Group SD5	SD7	*P* value
	(*n* = 25)	(*n* = 24)	(*n* = 25)	
Time to awake (min)	17.6 ± 5.4	19.6 ± 5.7	20.2 ± 5.7	0.24
Rescue analgesia (patients)	18 (72.0)	5 (20.8)	4 (16.0	0.0005
Incidence of PONV	12 (48.0)	4 (16.7)	4 (16.0)	0.015

Data are expressed as mean ± standard deviation (One-way ANOVA test) or number of patients (percentage) (Chi-Square test)

*SR* sevoflurane-remifentanil group, *SD5* sevoflurane-dexmedetomidine group (0.5 μg/kg), *SD7* sevoflurane-dexmedetomidine group (0.7 μg/kg), *PONV* postoperative nausea and vomiting

## Discussion

The main result of our study was that during sevoflurane-based anesthesia, dexmedetomidine infusion 0.7 μg/kg administered over 10 min at the end of surgery produced a dose-dependent effect in providing smooth extubation without significantly prolonging recovery from anesthesia. Compared to remifentanil, the benefits of dexmedetomidine included hemodynamic stability, opioid sparing, and less PONV. The disadvantages of dexmedetomidine included lower tidal volume and blood pressure.

If endotracheal intubation is utilized, smooth emergence from anesthesia may be a challenge in many patients undergoing otologic surgery. Laryngeal responses, with bucking and coughing on emergence from anesthesia, are undesirable, since the pressure transmitted through the eustachian tubes may unseat a tympanic membrane graft or disrupt other repairs [15]. Accordingly, many anesthesiologists prefer to perform tracheal extubation when the spontaneously breathing patient remains at a deep plane of anesthesia [1]. A key aspect of the deep extubation technique is for airway reflexes to be effectively suppressed at the time of extubation, but resume as soon as possible after extubation. Because of remifentanil’s analgesic and antitussive properties, the combination of sevoflurane and remifentanil [5, 16] is commonly used to decrease the MAC of sevoflurane and facilitate a more rapid recovery.

Dexmedetomidine, like remifentanil, has been shown to decrease sevoflurane MAC [7, 17]. Several studies have confirmed the efficacy of both remifentanil and dexmedetomidine for airway reflex suppression during flexible bronchoscopy and awake intubation [7, 8]. In addition, Guler et al. [10] reported that a single dose of dexmedetomidine (0.5 μg/kg) before the end of the surgery attenuated airway reflexes during extubation. In the current study, when combined with 1 MAC sevoflurane, both dexmedetomidine 0.7 μg/kg and remifentanil effectively suppressed airway reflexes and facilitated smooth extubation in deeply anesthetized patients. Compared to Group SD5, the higher dose of dexmedetomidine in Group SD7 was associated with a higher percentage of patients with a smooth extubation. In addition, compared to Group SR and Group SD5, patients receiving dexmedetomidine 0.7 μg/kg did not exhibit a prolonged time to wake.

If general endotracheal anesthesia is used during otologic surgery, a deep stage of anesthesia is required until the end of procedure to avoid bucking and coughing associated with head movement during application of the surgical dressing. In the current study, the majority of patients in all groups maintained a regular respiratory pattern during dressing application and tracheal tube cuff deflation, and when patients maintained a regular respiratory pattern during dressing application, tracheal extubation could be performed smoothly in all cases. It is generally accepted that the absence of a reaction and maintenance of regular respirations during tracheal tube cuff deflation is a reliable predictor of a smooth deep extubation [18]. In our experience, airway stimulation is greater during dressing application than during cuff deflation. Thus, a regular respiratory pattern during dressing application is a more reliable predictor of smooth tracheal extubation in spontaneously breathing anesthetized patients after otologic surgery.

In the current study, the respiratory rate during tracheal extubation was lower in Group SR than in Group SD5 or Group SD7. This result was consistent with the known respiratory depressant effects of remifentanil, which does not occur with dexmedetomidine. However, there was an exception, as one spontaneously breathing patient in Group SD5 exhibited apnea during dexmedetomidine infusion. The underlying reason for this was unclear. Moreover, the mean tidal volume was lower in patients receiving dexmedetomidine. We speculated that in presence of sevoflurane, the respiratory depression caused by sevoflurane and dexmedetomidine may reflect decrease in central respiratory drive mediated by both GABA_A_ and alpha-2 adrenergic receptors. The potential disadvantages of dexmedetomidine are hemodynamic changes, such as decrease in heart rate and/or increased or decreased blood pressure, but those adverse effects can be prevented by administering dexmedetomidine over 10 min. In our study, hemodynamic effects were clinically insignificant in patients receiving dexmeedetomidine.

Another disadvantage of remifentanil is its ultrashort duration of analgesia. Our findings were consistent with this disadvantage. For example, the increase in MAP and HR after extubation reflected rapid dissipation of the effects of remifentanil. Furthermore, morphine for rescue analgesia in the PACU was required by 18 patients in Group SR, but only by 5 and 4 patients in Groups SD5 and SD7, respectively. In addition, one 18-year-old patient in Group SR experienced agitation. When he regained full consciousness, he reported bad dreams and requested analgesia.

Arsian et al. [19] reported that the incidence of PONV for patients undergoing otologic surgery was as high as 65.7 %, which could be reduced to 22.9 % by the use of prophylactic antiemetic drugs. Apfel et al. [20] recommended first-line strategy with dexamethasone and a 5-HT receptor antagonist to prevent PONV. In our current study, we found that fewer patients in Group SD5 and Group SD7 reported PONV on the first postoperative day, compared to patients in Group SR. This may be attributable to the opioid-sparing and antiemetic properties of dexmedetomidine.

The potential risks of extubation at a deep level of anesthesia include aspiration, airway obstruction, desaturation, and loss of airway control. In our study, we did not observe aspiration, severe laryngospasm, or desaturation in any of the three groups from the time of tracheal extubation to full recovery. Several factors likely contributed to these findings. First, the patients in our study were well fasted. Second, we used relatively short-acting anesthetic agents and adjuncts, including fentanyl, sevoflurane, and remifentanil. We also used the short-acting muscle relaxant mivacurium and objectively confirmed the return of neuromuscular function. Third, patients who experienced a smooth extubation maintained a regular respiratory pattern during dressing application, a maneuver that produced major stimulation of the airway. Fourth, the oropharynx was well suctioned before extubation.

There are several limitations in our study. First, during extubation at a deep level of anesthesia, we placed the patient in a supine instead of lateral position. Our rationale for this position was that if loss of the airway occurred, the anesthesiologist could more quickly manage the airway when patients were supine. Second, we monitored anesthesia depth by clinical signs rather than the bispectral index (BIS). We acknowledge that BIS may have provided useful information, but BIS monitoring was not routinely available at our hospital and we wanted our study to reflect usual clinical conditions. Third, this study involved highly selected, healthy patients undergoing middle ear surgery, who had no history or physical characteristics suggesting potential airway difficulties. Thus, it is uncertain whether our results can be generalized to other populations. Fourth, Insommo [11] suggested that tracheal extubation could be performed at nearly 1 MAC sevoflurane. However, we have found that with the anesthesia technique described in the Materials section of that study, we are unable to achieve a smooth extubation for patients after otologic surgery. Instead, we routinely perform tracheal extubation at either 1.3 MAC sevoflurane anesthesia or at 1 MAC sevoflurane in combination with low-dose remifentanil, as was used in this study [5]. Fifth, evaluation of smooth extubation was limited in the first 1 min after deep extubation. 1 min is a narrow interval. Though respiratory complication usually happens immediately after extubation, patients should be closely monitored during emergence.

## Conclusion

In summary, our study showed that similar to remifentanil, dexmedetomidine 0.7 μg/kg produced smooth tracheal extubation in adult patients deeply anesthetized with sevoflurane after otologic surgery. In addition, dexmedetomidine exhibited opioid-sparing effects and produced less PONV than remifentanil.

## Abbreviations

MAC: Minimum alveolar concentration
ETCO2: End-tidal carbon dioxide
PONV: Postoperative nausea and vomiting
VAS: Visual analogue scale
PACU: Post-anesthesia care unit.
